# Association of Acquired and Heritable Factors With Intergenerational Differences in Age at Symptomatic Onset of Alzheimer Disease Between Offspring and Parents With Dementia

**DOI:** 10.1001/jamanetworkopen.2019.13491

**Published:** 2019-10-16

**Authors:** Gregory S. Day, Carlos Cruchaga, Thomas Wingo, Suzanne E. Schindler, Dean Coble, John C. Morris

**Affiliations:** 1 Charles F. and Joanne Knight Alzheimer Disease Research Center, Washington University School of Medicine in St Louis, St Louis, Missouri; 2 Department of Neurology, Washington University School of Medicine in St Louis, St Louis, Missouri; 3 Department of Psychiatry, Washington University School of Medicine in St Louis, St Louis, Missouri; 4 Department of Neurology, Emory University, Atlanta, Georgia; 5 Department of Human Genetics, Emory University, Atlanta, Georgia; 6 Department of Biostatistics, Washington University School of Medicine in St Louis, St Louis, Missouri

## Abstract

**Importance:**

Acquired and heritable traits are associated with dementia risk; however, how these traits are associated with age at symptomatic onset (AAO) of Alzheimer disease (AD) is unknown. Identifying the associations of acquired and heritable factors with variability in intergenerational AAO of AD could facilitate diagnosis, assessment, and counseling of the offspring of parents with AD.

**Objective:**

To quantify the associations of acquired and heritable factors with intergenerational differences in AAO of AD.

**Design, Setting, and Participants:**

This nested cohort study used data from the Knight Alzheimer Disease Research Center that included community-dwelling participants with symptomatic AD, parental history of dementia, and available DNA data who were enrolled in prospective studies of memory and aging from September 1, 2005, to August 31, 2016. Clinical, biomarker, and genetic data were extracted on January 17, 2017, and data analyses were conducted from July 1, 2017, to August 20, 2019.

**Main Outcomes and Measures:**

The associations of acquired (ie, years of education; body mass index; history of cardiovascular disease, hypertension, hypercholesterolemia, diabetes, active depression within 2 years, traumatic brain injury, tobacco use, and unhealthy alcohol use; and retrospective determination of AAO) and heritable factors (ie, ethnicity/race, paternal or maternal inheritance, parental history of early-onset dementia, *APOE *ε4 allele status, and AD polygenic risk scores) to intergenerational difference in AAO of AD were quantified using stepwise forward multivariable regression. Missense or frameshift variants within genes associated with AD pathogenesis were screened using whole-exome sequencing.

**Results:**

There were 164 participants with symptomatic AD, known parental history of dementia, and available DNA data (mean [SD] age, 70.9 [8.3] years; 90 [54.9%] women) included in this study. Offspring were diagnosed with symptomatic AD a mean (SD) 6.1 (10.7) years earlier than their parents (*P* < .001). The adjusted *R*^2^ for measured acquired and heritable factors for intergenerational difference in AAO of AD was 0.29 (*F*_8,155_ = 9.13; *P* < .001). Paternal (β = −9.52 [95% CI, −13.79 to −5.25]) and maternal (β = −6.68 [95% CI, −11.61 to −1.75]) history of dementia, more years of education (β = −0.58 [95% CI −1.08 to −0.09]), and retrospective determination of AAO (β = −3.46 [95% CI, −6.40 to −0.52]) were associated with earlier-than-expected intergenerational difference in AAO of AD. Parental history of early-onset dementia (β = 21.30 [95% CI, 15.01 to 27.59]), presence of 1 *APOE *ε4 allele (β = 5.00 [95% CI, 2.11 to 7.88]), and history of hypertension (β = 3.81 [95% CI, 0.88 to 6.74]) were associated with later-than-expected intergenerational difference in AAO of AD. Missense or frameshift variants within genes associated with AD pathogenesis were more common in participants with the greatest unexplained variability in intergenerational AAO of AD (19 of 48 participants [39.6%] vs 26 of 116 participants [22.4%]; *P* = .03).

**Conclusions and Relevance:**

Acquired and heritable factors were associated with a substantial proportion of variability in intergenerational AAO of AD. Variants in genes associated with AD pathogenesis may contribute to unexplained variability, justifying further study.

## Key Points

**Question:**

What are the associations of acquired and heritable factors with intergenerational differences in age at symptomatic onset (AAO) of Alzheimer disease (AD) among offspring of parents with AD?

**Findings:**

In this cohort study including 164 participants with symptomatic AD and a parental history of dementia, the factors of parental inheritance, more years of education, and retrospective determination of AAO were associated with an earlier-than-expected AAO of AD; parental history of early-onset dementia, *APOE *ε4 allele status, and hypertension were associated with a later-than-expected AAO of AD. Missense or frameshift variants within genes associated with AD pathogenesis were more common in participants with greater unexplained variability in intergenerational AAO of AD.

**Meaning:**

Acquired and heritable factors were associated with a substantial proportion of variability in intergenerational AAO of AD.

## Teaser

This cohort study examines the associations of acquired and heritable factors associated with intergenerational differences in age at symptomatic onset of Alzheimer disease among offspring of parents with Alzheimer disease.

## Introduction

After age, genetic factors are the second-greatest factors associated with risk of symptomatic Alzheimer disease (AD). Therefore, it is not surprising that offspring of parents with dementia have an increased lifetime risk of symptomatic AD,^1,2^ with the greatest risk experienced by those with a maternal history or with 2 affected parents^3,4,5^ and those who inherited 1 or 2 copies of the *APOE* (OMIM 107741) ε4 allele.^6^ Prior studies have suggested that disease expression may change across generations, with the offspring of affected parents experiencing an earlier age at symptomatic onset (AAO) of AD.^1,2,7,8^ A 2017 study by Livingston et al^9^ suggested that the cumulative effects of acquired and heritable traits are associated with intergenerational dementia risk. However, how these traits are associated with AAO of AD is unknown, to our knowledge. Identifying the associations of acquired and heritable factors with variability in intergenerational AAO of AD may facilitate diagnosis, assessment, and counseling of offspring of affected parents who have a high risk of developing dementia and high interest in knowing their dementia risk.^10^ These same factors may also be exploited to delay onset of AD and improve patient outcomes.

We evaluated the associations of well-characterized acquired and heritable risk and protective factors for AD to variability in intergenerational AAO in people whose parents had dementia and who were enrolled in Knight Alzheimer Disease Research Center (ADRC) of Washington University in St Louis, St Louis, Missouri, longitudinal studies of memory and aging. We hypothesized that measured factors would be associated with a significant proportion of observed variability. Furthermore, we considered whether offspring with the greatest unexplained variability might have additional genetic variants that were associated with variability in intergenerational AAO of AD. In this way, we used variability in intergenerational AAO as a putative endophenotype to identify individuals who may have genetic variants that accelerate or delay onset of AD.

## Methods

### Participant Identification and Recruitment

The Knight ADRC recruits and longitudinally assesses community-dwelling adults older than 45 years via prospective studies of memory and aging. Eligible participants may be asymptomatic or have early symptoms of AD. All participants are required to have an observant informant who can provide collateral history and are asked to participate in core study procedures, including longitudinal clinical assessments, neuropsychological testing, neuroimaging, and biofluid biomarker studies.

Participants in this study were enrolled within specific longitudinal Knight ADRC studies (ie, Adult Children Study or Healthy Aging and Senile Dementia Project) from December 4, 1979, to August 31, 2016, and assessed according to standard Knight ADRC practices. Research nurses or social workers reviewed medical and family history at enrollment and subsequent visits. Parental AAO of dementia was reported by study participants and verified with study partners and family members. All participants were assessed annually by experienced clinicians using a semistructured interview with a knowledgeable collateral source and the symptomatic individual and by detailed neurological examination. A clinical diagnosis of dementia was considered by study clinicians at the conclusion of each assessment, integrating results from the clinical assessment and bedside measures of cognitive function (detailed elsewhere^11^). Dementia diagnostic criteria conformed to National Institute on Aging–Alzheimer’s Association Work Group recommendations.^12^ Dementia stages were classified using the global Clinical Dementia Rating and assigned by qualified clinicians in accordance with established scoring rules.^13^ Participants from families with known disease-causing *APP* (OMIM 104760), *PSEN1* (OMIM 104311), and *PSEN2* (OMIM 600759) mutations were excluded.

Participants with a clinical diagnosis of symptomatic AD and reported parental history of dementia were selected through review of the Knight ADRC database. To ensure uniform collection and reporting of variables of interest for subsequent analyses, the data set was limited to participants who were evaluated after the Uniform Data Set version 1.2 was introduced on September 1, 2005,^14^ and from whom DNA data were available. In a few instances, the parents of participants were also longitudinally studied, providing an opportunity to compare reported and observed parental AAO. All participants or their delegates provided written informed consent or assent to the use of clinical and genetic information for research purposes. The Washington University School of Medicine Institutional Review Board approved all study procedures. Clinical, biomarker, and genetic data were extracted on January 17, 2017. Data were analyzed from July 1, 2017, to August 20, 2019. Results were reported in accordance with the Strengthening the Reporting of Observational Studies in Epidemiology (STROBE) reporting guideline for cohort studies.

### Data Collection and Analyses

Participants self-reported Hispanic or Latino ethnicity and race (ie, white, African American, American Indian, Alaskan Native, Native Hawaiian or other Pacific Islander, Asian, other, or unknown). Acknowledging the putative associations of ethnicity and race to differences in AD,^1^ reported African American ancestry, the minority racial group most commonly represented in our cohort, was included in multivariable analyses. Lifetime health history was obtained from participants and collateral sources at each assessment, including questions assessing interval health, medication use, and personality or behavioral changes (including mood). Participants’ weight and height were measured at each visit, and body mass index was derived, calculated as weight in kilograms divided by height in meters squared. When multiple assessments were completed, variables measured at the clinical assessment closest to the time of dementia diagnosis (Clinical Dementia Rating ≥0.5) were used in analyses. Information concerning the prevalence and severity of obstructive sleep apnea was not collected as a part of the Uniform Data Set until 2016^15^; therefore, obstructive sleep apnea was not considered as a variable. A detailed description of variables is provided in eAppendix 1 in the Supplement.

For participants who did not have symptoms of dementia at enrollment, AAO was prospectively defined as the age when the global Clinical Dementia Rating was 0.5 or higher. For participants who had symptomatic AD at enrollment, AAO was retrospectively determined through an interview with a reliable collateral source. The difference in AAO of AD between parent and offspring was derived for each participant-parent dyad by subtracting parental AAO from participant AAO. When both parents were affected, we used the mean parental AAO to determine the difference in AAO. In this way, we derived a continuous measure of disparity in intergenerational AAO of AD, identifying participants with earlier-than-expected (negative difference in AAO) and later-than-expected (positive difference in AAO) AAO of AD.

Cerebrospinal fluid (CSF) measures of amyloid-β peptide 42, total tau, and phosphorylated tau 181,^16^ or carbon 11–labeled Pittsburgh compound B or florbetapir amyloid positron emission tomography retention^17,18^ were obtained from patients for whom such data were available. Genome-wide association study data were quality controlled and imputed as previously reported.^19^ A weighted polygenic risk score (PRS) for AAO of AD was generated for the same participants,^19,20,21^ excluding *APOE* ε allele status, which was included in multivariable analyses. Known dementia-associated and novel genetic variants were screened in all participants within relevant genes (eg, *PSEN1*, *PSEN2*, *APP*, *TREM2* [OMIM 605086], *PLD3* [OMIM 615698], *MAPT* [OMIM 157140], *C9orf72* (OMIM 614260) and *GRN* [OMIM 607485]) using whole-exome sequencing. Databases detailing genes associated with genetic variants associated with AD, frontotemporal dementia, and Parkinson disease were used to annotate the whole-exome sequencing data, defining known pathogenic variants (ie, AD&FTD Mutation Database, Parkinson Disease Mutation Database).^22^ These databases are updated continuously with pathogenic and nonpathogenic genetic variants that occur in the coding regions of genes associated with AD, frontotemporal dementia, and Parkinson disease and are reported in the literature, reported at scientific meetings, or directly submitted. Genetic analyses and AD biomarker measures are detailed in eAppendix 2 in the Supplement.

### Statistical Analysis

Data were analyzed using SPSS statistical software version 24.0 (IBM). Groupwise differences for categorical variables were determined using the Fisher exact or Pearson χ^2^ tests. Continuous measures were compared using the *t* test unless otherwise stated. The associations of the intergenerational difference in AAO of AD (dependent variable) with acquired (ie, years of education; body mass index; history of cardiovascular disease, hypertension, hypercholesterolemia, diabetes, active depression within 2 years, traumatic brain injury, tobacco abuse, or unhealthy alcohol use; or retrospective determination of AAO) and heritable factors (ie, ethnicity/race, paternal or maternal history of dementia, parental history of early-onset dementia, *APOE *ε4 allele status, or AD PRS) were explored using stepwise multivariable linear regression (α = .05 for entry; α = .10 for removal), controlling for age and sex (forced entry). To assess for collinearity, variables of interest were included in a linear regression (forced entry) model and variance inflation factor quotients were computed. Variance inflation factors less than 5 were considered to indicate low probability of collinearity. Model explanatory power and fit were assessed using the adjusted *R*^2^ and analysis of variance. Residual values were determined on a participant-by-participant basis by subtracting the expected intergenerational difference in AAO of AD (ie, that predicted from the model) from the observed intergenerational difference in AAO so that negative residuals identified participants whose observed intergenerational difference in AAO occurred earlier than expected and positive residuals participants whose intergenerational difference in AAO of AD occurred later than predicted. *Z* scores of residuals were determined, and participants were rank ordered. Participants with greater-than-expected residuals (ie, highest variability in intergenerational difference in AAO of AD not associated with measured variables) were defined as those with residuals more than 1 SD below (risk) or above (resilience) the mean. *P* values were 2-tailed, and statistical significance was defined as *P* less than .05 and Bonferoni corrected where appropriate.

## Results

**Figure 1.**
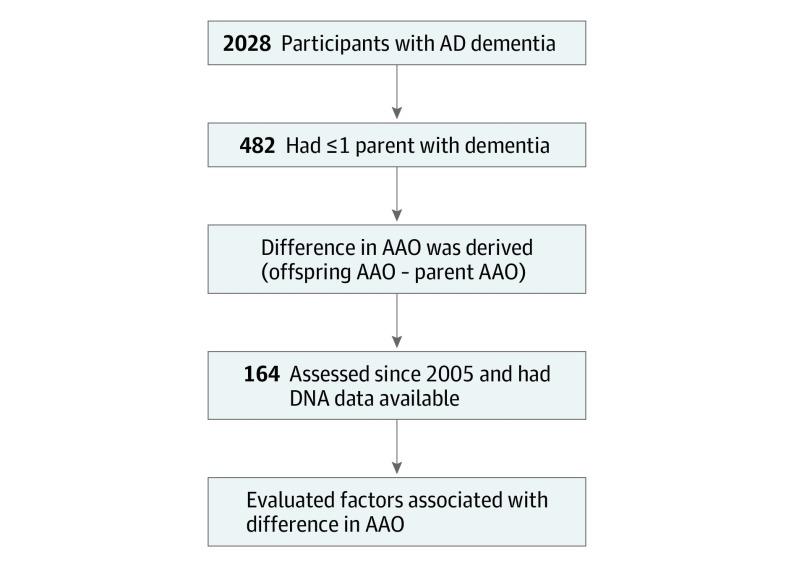
Participant Selection AAO indicates age at symptomatic onset; AD, Alzheimer disease.

Of 2028 Knight ADRC participants with symptomatic AD, 482 (23.7%) had at least 1 parent with dementia and reported AAO of AD. Of these, 164 participants (34.0%) were evaluated after 2005, had DNA available, and were included in this study (Figure 1). The mean AAO of AD among the full cohort was 70.9 (8.3) years, and 90 (54.9%) were women. At study inclusion, enrolled participants had completed a mean (SD) of 3.5 (4.6) annual clinical assessments (range, 1-27). Biomarkers were consistent with AD in 84 of 98 offspring of affected parents (85.7%) for whom biomarker data were available. No statistically significant differences were found between participants in whom biomarker data was consistent vs inconsistent with AD as the etiologic cause of dementia (eTable 1 in the Supplement). Accordingly, all eligible participants were included in analyses, consistent with our objective to characterize the associations of acquired and heritable factors with intergenerational variability in AAO among offspring who had received clinical diagnoses of symptomatic AD and their parents with AD.

Demographic characteristics and clinically relevant symptoms and signs are summarized in Table 1. The cohort included 102 participants who were symptomatic at the time of study enrollment, and their AAO of AD was derived from retrospective report. This group exhibited a younger mean (SD) AAO than the 62 participants who developed dementia during prospective follow-up and whose AAO was determined via prospective observation (mean [SD] AAO, 69.1 [7.4] years; range, 46-87 years vs 73.8 [8.9] years; range, 55-96 years; mean difference, −4.7 [95% CI, −7.2 to −2.1]; *P* < .001), suggesting that prospective follow-up did not result in earlier recognition of AD onset. Uniquely, the parents of 11 participants had also participated in longitudinal studies of memory and aging at the Knight ADRC, with AAO prospectively determined under near-identical protocols. No differences were observed (mean [SD] AAO, 76.7 [10.0] years; range, 60-95 years) vs retrospectively reported (mean [SD] AAO, 76.4 [9.8] years; range, 60-92 years) AAO in these parents (Wilcoxon signed rank test: *P* = .52; *Z* = −0.65). The parents of all 11 offspring had received clinical diagnoses of probable AD, which were confirmed in all 8 parents who underwent autopsy.

**Table 1.** Participant Characteristics at the Time of Diagnosis With Symptomatic AD

Characteristic	No. (%) (N = 164)
AAO of AD, mean (SD) [range], y	70.9 (8.3) [46-96]
Women	90 (54.9)
Education, mean (SD) [range], y	15.4 (2.9) [8-29]
Race/ethnicity	
Non-Hispanic white	144 (87.8)
African American	19 (11.6)
Asian	1 (0.6)
*APOE *ε4 allele carriers	110 (67.1)
1 copy (ε2/4, ε3/4)	89 (54.3)
2 copies (ε4/4)	21 (12.8)
Global Clinical Dementia Rating, median (range)	0.5 (0.5-1.0)
Mini-Mental State Examination Score, mean (SD) [range]	25.0 (3.9) [9-30]
Parental AAO of AD, mean (SD) [range], y	
Maternal	77.3 (10.4) [39-102]
Paternal	77.5 (10.7) [47-97]

Abbreviations: AAO, age at symptomatic onset; AD, Alzheimer disease.

**Figure 2.**
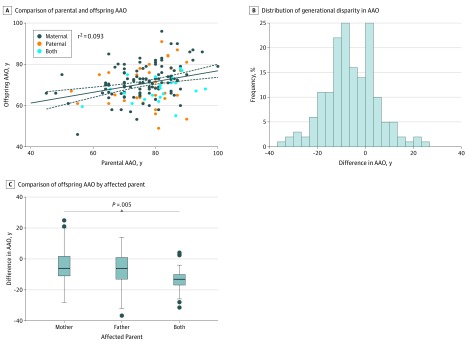
Association of Age at Symptomatic Onset (AAO) of Alzheimer Disease in Parents and Offspring A, The solid line indicates the *r*^2^ regression line; dashed lines, 95% CI. C, Circles indicate participants whose difference in AAO was beyond the interquartile range; center horizontal line, median; top and bottom borders of box, 75% and 25% values of interquartile range, respectively; and whiskers, interquartile range.

In the full cohort, a modest correlation was observed between parental and offspring AAO (*r*^2^ = 0.09; *F*_1,162_ = 16.6; *P* < .001; Figure 2A), with symptoms of AD developing a mean (SD) of 6.1 (10.7) years earlier in offspring compared with their parent (*t* = −7.3; *df* = 163; *P* < .001). A maternal history of AD was most common, reported by 108 offspring (65.8%). Paternal inheritance was reported by 35 offspring (21.3%). Both parents were affected for 21 offspring (12.8%). Offspring with 2 affected parents had a mean difference in AAO of 8.0 (95% CI, 2.0-14.0) years earlier than those with just an affected mother (*P* = .005) and 6.0 years (95% CI, 1.0-13.0) earlier than those with just an affected father (*P* = .11) (Figure 2C).

The association of acquired and heritable factors with intergenerational difference in AAO of AD was quantified using stepwise multivariable linear regression controlling for sex and years of education. The adjusted *R*^2^ of acquired and heritable risk factors for intergenerational variability in AAO of AD was 0.29 (*F*_8,155_ = 9.13; *P* < .001). Earlier-than-expected intergenerational difference in AAO was associated with paternal (β = −9.52 [95% CI, −13.79 to −5.25]) and maternal (β = −6.68 [95% CI, −11.61 to −1.75]) history of dementia, having more years of education (β = −0.58 [95% CI, −1.08 to −0.09]), and retrospective reporting of AAO of AD (β = −3.46 [95% CI, −6.40 to −0.52]). Later than expected intergenerational difference in AAO was associated with parental history of early-onset dementia (β = 21.30 [95% CI, 15.01-27.59]), presence of 1 *APOE *ε4 allele (β = 5.00 [95% CI, 2.11-7.88]), and history of hypertension (β = 3.81 [95% CI, 0.88-6.74]) (Table 2). Variance inflation factors were low (range, 1.13-2.32), suggesting that these results were not substantially influenced by collinearity. The addition of polygenic risk scores specific to AAO of AD did not improve model performance (adjusted *R*^2^ change = 0.001; *F*_1,114_ change = 0.11; *P* = .74). Similar weights were established when analyses were limited to 84 participants with biomarker-confirmed AD (adjusted *R*^2^ = 0.33; *F*_8,75_ = 6.18; *P* < .001) (eTable 2 in the Supplement). Post hoc analyses considering participant AAO independent of parental AAO confirmed the expected association of *APOE* genotype with AAO of AD (eTable 3 in the Supplement).^23,24^ *APOE *ε4/4 carrier status was associated with an earlier-than-expected AAO (β = −5.87 [95% CI, −9.25 to −2.50]; *P* = .001) after controlling for other acquired and heritable factors (eTable 4 in the Supplement).

**Table 2.** Results of Multivariate Stepwise Linear Regression of Associations of Measured Factors With Intergenerational Difference in AAO of Alzheimer Disease

Factor	*β* (95% CI)	*P* Value
Intercept	8.38 (−2.02 to 18.78)	.11
Forced entry		
Female	−1.13 (−4.11 to 1.86)	.46
Education, mean (SD), y	−0.58 (−1.08 to −0.09)	.02
Selected factors		
Father affected	−9.52 (−13.79 to −5.25)	<.001
Mother affected	−6.68 (−11.61 to −1.75)	.01
Parent with early-onset dementia	21.30 (15.01 to 27.59)	<.001
History of hypertension	3.81 (0.88 to 6.74)	.01
*APOE *ε4/2 or ε4/3	5.00 (2.11 to 7.88)	.001
Symptomatic at study entry (retrospective determination of AAO)	−3.46 (−6.40 to −0.52)	.02
Excluded factors		
Active depression within 2 y	−0.12 (NA)	.07
History of cardiovascular disease	0.10 (NA)	.15
History of tobacco use, >30 pack-years^a^	0.10 (NA)	.15
History of hypercholesterolemia	−0.11 (NA)	.15
Body mass index	−0.06 (NA)	.35
History of diabetes	−0.06 (NA)	.37
African American race	−0.05 (NA)	.45
* APOE *ε4/4	−0.04 (NA)	.60
History of unhealthy alcohol use	−0.02 (NA)	.74
History of traumatic brain injury	−0.01 (NA)	.91

Abbreviations: AAO, age at symptomatic onset; NA, not applicable.

^a^ Calculated as packs of cigarettes used per day × number of years of use.

We further considered the association of additional unmeasured heritable factors with unexplained intergenerational variability in AAO of AD using whole-exome sequencing to investigate for known and potentially novel missense or frameshift variants within genes associated with AD and AD-related dementia pathogenesis. Sixty-four missense or frameshift variants in *PSEN1, PSEN2, APP, TREM2, PLD3, MAPT*, *C9orf72* and *GRN* were identified in 45 offspring (27.4%). Low-frequency coding variants were detected more frequently in participants with greater unexplained intergenerational variability in AAO compared with participants with less unexplained variability (19 of 48 offspring [39.6%] vs 26 of 116 offspring [22.4%]; odds ratio, 2.27 [95% CI, 1.10-4.68]; *P* = .03), raising the possibility that these variants were associated with disparity in intergenerational AAO. Only 2 such variants have previously been associated with an increased risk of AD in the AD&FTD Mutation Database and Parkinson Disease Mutation Database^25^: (1) *TREM2*, p.Arg47His, detected in 2 participants with later-than-expected intergenerational difference in AAO and 1 participant with earlier-than-expected intergenerational difference in AAO; and (2) *PLD3*, p.Val232Met, detected in 1 participant with later-than-expected intergenerational difference in AAO. Table 3 lists the variants discovered in participants with the highest unexplained intergenerational variability in AAO.

**Table 3.** Variants Identified Predominantly in Participants With the Greatest Unexplained Intergenerational Variability in AAO of Alzheimer Disease^a^

*Z* Score of Residuals	Gene (Variant) [Amino Acid Position]	Mean Residual (Range)^b^
<−1 SD, earlier-than-expected offspring AAO	*GRN* (42429501:G:A) [p.Arg433Gln]	−1.63
	MAPT (44061123:C:T) [p.Ser318Leu]	−1.56 (−2.27 to −0.77)
	*PLD3* (408758:G:A) [p.Val159Met]	−1.38
	*PSEN1* (73673178:A:G) [p.Glu318Gly]	−1.04 (−2.13 to 0.64)
>1 SD, later-than-expected offspring AAO	*GRN* (42429835:G:A) [p.Val514Met]	1.49
	*C9orf72*^c^	1.34
	*PSEN2* (A346S)	1.32
	*PLD3* (40877595:G:A) [p.Val232Met]	1.31
	*APP* (V287G)	1.11

Abbreviation: AAO, age at symptomatic onset.

^a^ Residual values from the model were normalized; high variability was defined as a *Z* score of residual SD less than −1 or greater than 1.

^b^ Items without a range were reported in only 1 participant.

^c^ Twenty-nine repeats.

## Discussion

A parental history of dementia was common in participants with symptomatic AD enrolled in longitudinal studies of memory and aging at the Knight ADRC , emphasizing the important associations of family history with AD risk.^1,2,4^ However, the correlation between AAO in parents and offspring was modest (*r*^2^ = 0.09), in contrast to that observed in a 2014 study by Ryman et al^26^ of families with autosomal dominant AD, in whom a substantial proportion of variance in AAO was explained by parental AAO (*r*^2^ = 0.38). This finding suggests that other factors may modulate AAO within families without autosomal dominant AD. Indeed, our results suggest that acquired and heritable factors were associated with a significant proportion of variability in intergenerational AAO (adjusted *R*^2^ = 0.29) with a bias toward an earlier-than-expected intergenerational difference in AAO in successive generations. This anticipation effect was most apparent in the offspring of 2 affected parents, which is consistent with prior findings in first-degree relatives of individuals with late-onset AD.^1,2,4,7,8^ Beyond parental inheritance, more years of education and retrospective reporting of AAO were also associated with an earlier-than-expected intergenerational difference in AAO, while *APOE *ε4 heterozygosity, family history of early-onset dementia, and late-life hypertension were associated with later-than-expected intergenerational difference in AAO. If replicated in additional cohorts, this information may be leveraged to improve estimates of AAO of AD in the offspring of parents with AD, potentially influencing the timing of dementia screening in clinical and research settings, including clinical trials of putative AD-modifying therapies designed to prevent or slow the development of symptomatic AD in presymptomatic individuals.

Somewhat unexpectedly, the presence of an *APOE *ε4 allele in the offspring of affected parents was associated with a later-than-expected AAO compared with offspring without *APOE *ε4. While these findings appear to conflict with previous reports that suggested an association of *APOE *ε4 allele status and younger AAO,^23,24^ it is important to recognize that our study describes the association of *APOE *ε4 with intergenerational difference in AAO (ie, the difference between offspring and parental AAO of AD). In this context, the presence of an *APOE *ε4 allele in offspring was associated with a lower difference in AAO, counterbalancing the overall association of an earlier-than-expected AAO of AD in the offspring of affected parents. Put another way, *APOE *ε4 allele carriers exhibited an AAO of AD closer to that reported in their parents than noncarriers after controlling for other acquired and heritable factors. This association makes sense assuming that *APOE *ε4 alleles were inherited from an affected parent and therefore represented a shared dementia risk factor. Thus, although the presence of *APOE *ε4 alleles was associated with an earlier-than-expected AAO vs absence of *APOE *ε4 alleles, the presence of an *APOE *ε4 allele is unlikely to explain the bias toward an earlier-than-expected AAO in offspring of affected parents observed in this study and others.^1,2,7,8^

The observed association of parental history of early-onset AD and later-than-expected intergenerational difference in AAO of AD was also unexpected. Our study design may have contributed to this association, as participants from families with known autosomal dominant AD-causing mutations were excluded from our cohort. Thus, parental history of early-onset AD may have been associated with the combination of deleterious acquired and heritable factors, which were attenuated in subsequent generations, resulting in an AAO that more closely approximated the mean or expected AAO (ie, regression toward the mean). Indeed, studies considering first-degree relatives of individuals with sporadic early-onset dementia did not report a close concordance between AAO across affected first-degree relatives.^27,28^ Therefore, in families without known AD-causing mutations, a parental history of early-onset dementia may be associated with an increased lifetime risk of dementia without any association with AAO of AD.^29^

Several studies have suggested that cumulative years of education may be inversely associated with dementia risk,^9,30,31,32^ an observation thought to reflect cognitive reserve.^33^ This association is most consistently demonstrated in individuals with low (eg, <10 years) educational attainment.^9^ Less is known concerning the association of dementia risk and education beyond secondary school. To our knowledge, few studies have considered the association of education with AAO of AD in highly educated individuals, such as those included in this study. A 1999 study that did report on this reported a similar association to that observed here, with increasing years of education associated with an earlier-than-expected AAO.^34^ This observation may reflect earlier recognition of cognitive impairment in highly educated individuals. Future studies including participants with a broader range of educational attainment may yield different results.

The association of late-life hypertension with later-than-expected intergenerational difference in AAO of AD in our sample suggests that late-life hypertension may delay the symptomatic onset of AD. This finding may be interpreted as consistent with prior studies reporting an association of low blood pressure (especially low diastolic blood pressure) with higher risk of dementia in older individuals.^35,36,37,38^ Alternatively, receiving a diagnosis of late-life hypertension may prompt recognition and management of other vascular risk factors, mitigating intergenerational differences in AAO through management of other medical conditions known to be associated with dementia risk.^9,39,40^ This latter suggestion may explain the lack of association observed in intergenerational difference in AAO of AD with diabetes, hypercholesterolemia, smoking, and cardiovascular disease in our cohort. Future studies are needed to clarify the associations of these potentially modifiable risk factors with AAO of AD and the underlying mechanisms.

Importantly, these associations were found after controlling for retrospective vs prospective reporting of AAO of AD. Indeed, intergenerational difference in AAO occurred 3.46 (95% CI, −6.40 to −0.52) years earlier in participants who were symptomatic at study onset vs those who were diagnosed with dementia during prospective follow-up after controlling for all other variables. This observation may reflect greater interest in study participation among offspring with an AAO earlier than that witnessed in their parents, inaccuracy in reporting of AAO, or some combination of these ascertainment and recall biases. Regardless of the mediators of this association, this finding emphasizes the importance of controlling for systematic biases that may be associated with AAO of AD in studies enrolling symptomatic and asymptomatic participants.

Even after accounting for known acquired and heritable factors, most variability in AAO between parents and offspring was unaccounted for, implying that other factors were associated with intergenerational difference in AAO. We explored this possibility further, arguing that offspring with greater unexplained variability in intergenerational AAO of AD would be more likely to harbor genetic variants that were associated with intergenerational difference in AAO. Consistent with this hypothesis, low-frequency missense or frameshift coding variants within relevant genes (eg, *PSEN1*, *PSEN2*, *APP*, *TREM2*, *PLD3*, *MAPT*, *GRN*) were more common in offspring with the greatest unexplained variability in AAO of AD. Although most of the variants discussed in this study have not previously been associated with AD risk, the associations of these variants with AD pathogenicity should be evaluated in appropriate experimental models and in accordance with existing algorithms.^41^ Additionally, somatic variants or recombination or mosaicism within genes associated with AD pathogenesis may also be associated with intergenerational disparity in AAO, warranting further consideration in participants with greater unexplained intergenerational variability in AAO.^42,43^ In this way, measures of variability in intergenerational AAO may serve as putative endophenotypes associated with individuals who may have genetic variants that accelerate or delay onset of AD.

### Limitations

This study has limitations. Unmeasured nonphysiological factors likely contributed to intergenerational difference in AAO of AD in our cohort, including recall bias in estimates of parental AAO. In this context, it is reassuring that reported and observed parental AAOs were similar in the 11 offspring-parent dyads from whom these data were available. The lack of prospective information regarding parental health history, symptomatic course, and biomarker status presents an additional limitation. Longitudinal studies incorporating data from parents and offspring are needed to acquire this information, allowing diagnoses to be applied in accordance with evolving research criteria,^44^ and the association of shared and discordant acquired and heritable factors (including genetic variants) with AAO of AD to be determined in parents and offspring. Additionally, we acknowledge that approximately 15% of individuals from whom data were available did not have biomarker profiles consistent with AD. These findings are consistent with rates of clinical-pathologic discordance in other ADRC studies.^45^ Although the results of multivariable models were similar when limited to participants with biomarkers consistent with AD, the inclusion of participants without biomarker evidence of AD may have affected interpretation of study results. These collective concerns highlight the need to replicate study findings in additional well-characterized, prospectively evaluated cohort studies before applying these results outside of research settings.

## Conclusions

A substantial proportion of intergenerational variability in AAO of AD was associated with measured acquired and heritable factors in community-dwelling participants enrolled in longitudinal studies of memory and aging at the Knight ADRC. Variants in genes associated with AD pathogenesis may be also associated with a proportion of the intergenerational variability in AAO, justifying further study in appropriate cellular or animal models.

## Notes

Supplement. **eAppendix 1.** Definitions of Variables Used in Analyses **eAppendix 2.** Detailed Methods **eTable 1.** Participant Demographics at the Time of Diagnosis With Symptomatic AD Stratified by AD Biomarker Status **eTable 2.** Results of Multivariate Forward Linear Regression Quantifying the Contributions of Measured Factors to Difference in AAO of AD in Participants With Biomarker-Confirmed AD **eTable 3.** Effect of Measured Variables on Participant AAO **eTable 4.** Multivariate Stepwise Linear Regression Quantifying the Contributions of Measured Factors to Participant AAO **eReferences.**
