# Supplementary material for: Association of Acquired and Heritable Factors With Intergenerational Differences in Age at Symptomatic Onset of Alzheimer Disease Between Offspring and Parents With Dementia
Source: JAMA Netw Open. 2019 Oct 16;2(10):e1913491. doi: 10.1001/jamanetworkopen.2019.13491 (PMC6806434; doi:10.1001/jamanetworkopen.2019.13491)
Supplement: Supplement. — eAppendix 1. Definitions of Variables Used in Analyses eAppendix 2. Detailed Methods eTable 1. Participant Demographics at the Time of Diagnosis With Symptomatic AD Stratified by AD Biomarker Status eTable 2. Results of Multivariate Forward Linear Regression Quantifying the Contributions of Measured Factors to Difference in AAO of AD in Participants With Biomarker-Confirmed AD eTable 3. Effect of Measured Variables on Participant AAO eTable 4. Multivariate Stepwise Linear Regression Quantifying the Contributions of Measured Factors to Participant AAO eReferences. [file jamanetwopen-2-e1913491-s001.pdf]

Supplementary Online Content

Day GS, Cruchaga C, Wingo T, Schindler SE, Coble D, Morris JC. Association of acquired and heritable factors with intergenerational differences in age at symptomatic onset of Alzheimer disease between offspring and parents with dementia. *JAMA Netw Open*. 2019;2(10):e1913491. doi:10.1001/jamanetworkopen.2019.13491

eAppendix 1. Definitions of Variables Used in Analyses

eAppendix 2. Detailed Methods

eTable 1. Participant Demographics at the Time of Diagnosis With Symptomatic AD Stratified by AD Biomarker Status

eTable 2. Results of Multivariate Forward Linear Regression Quantifying the Contributions of Measured Factors to Difference in AAO of AD in Participants With Biomarker-Confirmed AD

eTable 3. Effect of Measured Variables on Participant AAO

eTable 4. Multivariate Stepwise Linear Regression Quantifying the Contributions of Measured Factors to Participant AAO

eReferences.

This supplementary material has been provided by the authors to give readers additional information about their work.

© 2019 Day GS et al. *JAMA Network Open.*

eAppendix 1. Definitions of Variables Used in Analyses

Detailed descriptions of parental, individual-specific, acquired and heritable factors included in analyses are included below. All variables were routinely assessed at annual clinical assessments, including detailed interview of the participant and collateral source. When multiple assessments were completed, variables measured at the clinical assessment closest to the time of dementia diagnosis (CDR ≥0.5) were reported and used in analyses.

<u>Parental factors</u>

Maternal history of dementia: Present (1) or absent (0). Detailed in family history documented at study enrollment.

Paternal history of dementia: Present (1) or absent (0). Detailed in family history documented at study enrollment.

Parental history of early-onset dementia: Present (1) or absent (0). Early-onset dementia was defined as present when reported age-at-symptomatic onset was <60 years.

<u>Individual-specific factors (demographics)</u>

Gender: Female (1) or male (0). Determined by participant self-report.

Education: Total years of formal education. E.g., completion of high school = 12 years of education.

Race: Participants self-reported Hispanic/Latino ethnicity and race (White, Black or African American, American Indian, Alaska Native, Native Hawaiian or other Pacific Islander, Asian, other, or unknown). Only African American race (1 present, 0 absent) was included in multivariable analyses.

<u>Acquired factors</u>

Body mass index: Derived for each participant, integrating measured height (cm) and weight (kg) in accordance with the following formula, (weight in kilograms) / (height in meters)^2.

History of cardiovascular disease: Present (1) or absent (0). Deemed present if participants reported a history of coronary artery disease, including history of percutaneous coronary intervention (stent), myocardial infarction, atrial fibrillation, congestive heart failure or pacemaker implantation.

History of hypertension: Present (1) or absent (0). Deemed present if participants reported a history of elevated blood pressure (systolic BP >140 mmHg, or diastolic BP >90 mmHg), requiring ongoing medical intervention (most commonly prescription of anti-hypertensive medication(s)).

History of hypercholesterolemia: Present (1) or absent (0). Deemed present if participants reported a history of elevated cholesterol, requiring ongoing medical intervention (most commonly prescription of a statin medication).

History of diabetes: Present (1) or absent (0). Deemed present if participants reported a history of type I or II diabetes mellitus, requiring ongoing medical intervention (most commonly prescription of anti-glycemic medications).

Active depression within 2 years: Present (1) or absent (0). Deemed present if the assessing clinician reported active (symptomatic) depression, or a history of depression within the past 2 years. Clinician assessment integrates information from the Geriatric Depression Scale, as well as participant and collateral source direct reporting concerning mood.

History of traumatic brain injury: Present (1) or absent (0). Deemed present if participant or collateral source endorsed a life-time history of traumatic brain injury associated with "brief" or "extended" loss of consciousness. Minor head injuries *not* resulting in loss of consciousness were scored as absent.

© 2019 Day GS et al. *JAMA Network Open.*

History of tobacco abuse: Present (1) or absent (0). Deemed present when participant or collateral source endorsed a lifetime tobacco use exceeding 30 "pack years" (# of packs per day x number of years of tobacco use).

History of alcohol abuse: Present (1) or absent (0). Deemed present when the participant or collateral source endorsed a history of prior or current alcohol abuse, where alcohol abuse was defined as a pattern of drinking "too much alcohol too often" that interferes with daily life.

Retrospective reporting of age-at-symptomatic onset (AAO): Present (1) or absent (0). Deemed present when the participant's AAO was determined by retrospective report of the participant and collateral source (versus diagnosis / designation during prospective follow-up).

<u>Heritable factors</u>

APOE ε4 allele status: Participants were defined according to the number of *APOE ε4* alleles: participants with one copy had genotypes *ε4/2, ε4/3*; participants with two copies had genotypes *ε4/4*. *APOE* genotyping was performed as previously described,[1] using DNA extracted from peripheral blood samples using standard procedures.

Polygenic AD risk score: Calculated as detailed in the manuscript Methods.

© 2019 Day GS et al. *JAMA Network Open.*

eAppendix 2. Detailed Methods

GWAS data

Knight ADRC participants were genotyped with the Illumina 660K, OmiExpress or Human Core exome. As part of routine quality control steps, single-nucleotide polymorphisms (SNPs) with minor allele frequency <1%, call rates <98%, Hardy–Weinberg equilibrium p-values $>10^{-6}$ and individuals with >2% missing genotypes were removed before imputation. Chromosome SNPs were analyzed to verify sex identification. Each genotyping array was imputed, separately, using SHAPEIT/IMPUTE2 with the 1000 Genomes Project as the reference panel. All genotypes with dosage levels <0.9 for all three possible genotypes or with information scores <0.3 were excluded. Variants out of Hardy–Weinberg equilibrium ($p<1x10^{-6}$) or with a genotyping rate below 95% were also omitted. After imputation all data from the different arrays were combined. Population structure was inferred by principal component (PC) analysis using PLINK v1.9. PLINK v1.9 was also used to find duplicate and related individuals (first cousins or more proximate) who were eliminated from the analyses. *APOE* ε2, ε3 and ε4 isoforms were determined by genotyping rs7412 and rs429358 using Taqman genotyping technology as previously described.[2]

Polygenic Risk Scores

Polygenic risk scores (PRS) were calculated as explained elsewhere.[3] Briefly, to derive the weighted PRS for AD-AAO, the odds ratios were modelled as reported in IGAP[4] using a logarithm of base 2 transformation. Single nucleotide polymorphisms (SNPs) that tag the genome wide signals from the IGAP were included (specific SNPs and odds ratios [OR] are listed in Cruchaga et al. 2018, supplementary Table 1[3]). SNPs utilized for the score either have a high genotyping rate (around 95%) or were a proxy to the IGAP hits (proxies included SNPs in high linkage disequilibrium [r^2>0.7] with the target SNP). PLINK v1.9 was used to calculate the PRS choosing the score function and the no-mean-imputation option to disallow imputed scores. The resulting mean was corrected by multiplying the allele count (log OR Score).

Whole Exome Sequencing

Exome libraries were prepared using Agilent's SureSelect Human All Exon kits V3 and V5 or Roche VCRome using a HiSeq2000 with paired end reads, with a mean depth of coverage of 50× to 150×. Alignment was conducted against GRCh37.p13 genome reference. Variant calling was performed following GATK's 3.6 Best Practices (https://software.broadinstitute.org/gatk/best-practices/) and restricted to Agilent's V5 kit plus a 100bp of padding added to each capture target end. We used BCFTOOLS (https://samtools.github.io/bcftools/bcftools.html) to decompose multiallelic variants into biallelic prior to variant quality control. Variant Quality Score Recalibration (VQSR) was performed separately for SNPs and INDELs. Only those SNPs and indels that fell above the 99.9 confidence threshold, as indicated by WQSR, were considered for analysis. Variants within low complexity regions were removed. Non-polymorphic variants and those outside the expected ratio of allele balance for heterozygosity calls (ABHet=0.3-0.7) were removed. Additional hard filters implemented included quality depth (QD ≥7 for indels and QD≥2 for SNPs), mapping quality (MQ≥40), fisher strand balance (FS≥200 for indels and FS≥60 for SNPs), Strand Odds Ratio (SOR≥10 for indels and SOR≥3 for SNPs), Inbreeding Coefficient (IC ≥-0.8 for indels) and Rank Sum Test for relative positioning of reference versus alternative alleles within reads (RPRS≥-20 for indels and RPRS≥-8 for SNPs). We used PLINK1.9 (https://www.cog-genomics.org/plink2/ibd) to remove variants that were out of Hardy–Weinberg equilibrium (p-value $<1\times10^{-6}$), with a genotype calling rate below 95%, with differential missingness between cases versus controls, WES vs WGS, or among different sequencing platforms (p-value$<1\times10^{-6}$).

AD biomarker data

CSF amyloid-β peptide 42 (Aβ42), total tau (tTau), and phosphorylated tau 181 (pTau) were measured with the corresponding Elecsys immunoassays on the Roche cobas e601 analyzer. Amyloid PET scans (^{11}C-Pittsburgh compound B; PiB) or Florbetapir (^{18}F-AV-45) were acquired on a Siemens Biograph mMR PET/MR scanner and attenuation corrected with a corresponding CT. Data were processed using an ROI approach using FreeSurfer software. Regional PIB or AV45 values were converted to standardized uptake value ratios (SUVRs) using cerebellar grey as a reference and partial volume corrected using a regional spread function approach.[5] Values from the left and right lateral orbitofrontal, medial orbitofrontal, precuneus, rostral middle frontal, superior frontal, superior temporal, and middle temporal cortices were averaged together to represent a mean cortical SUVR.

CSF biomarker positivity was defined as pTau/Aβ42 >0.0198,[6] which has very high concordance with amyloid PET positivity, defined as a mean cortical standardized uptake value ratio (SUVR) of >1.42 for PIB[7] and >1.219 for

© 2019 Day GS et al. *JAMA Network Open.*

AV45.[8] When results were discordant in participants with both amyloid PET and CSF AD biomarkers, amyloid PET findings were used.

© 2019 Day GS et al. *JAMA Network Open.*

eTable 1. Participant Demographics at the Time of Diagnosis With Symptomatic AD Stratified by AD Biomarker Status

Participant factors (n=96)	AD Biomarkers		p value†
	Positive (n=84)	Negative (n=14)	
Demographics			
AAO, mean ± SD, years	70.6±7.4	68.4±9.4	0.42
Female sex (%)	40 (48%)	8 (57%)	0.51
Education, mean ± SD (range), years	15.8±2.9 (12-29)	15.2±3.3 (12-23)	0.52
Ethnicity			
- Non-Hispanic White (%)	81 (96%)	13 (93%)	0.39
- African American (%)	2 (2%)	1 (7%)	0.37
- Other (%)	1 (<1%)	0	>0.99
Maternal AAO, mean ± SD, years	75.5±10.8	82.6±7.4	0.49
Paternal AAO, mean ± SD, years	75.5±12.1	77.3±9.9	0.39
Clinical information			
Global CDR, median (range)	0.5 (0.5-1.0)	0.5 (0.5-1.0)	0.70
Mini-Mental Status Examination score, mean ± SD (range)	25.4±3.6 (9-30)	26.4±3.0 (21-30)	0.70

Information was available for all variables (no missing data)
AAO=age-at-symptomatic onset; AD=Alzheimer disease.
†Student's t-test used for continuous variables; Chi-square test for categorical variables, except those with expected values <5 (Fisher Exact test)

© 2019 Day GS et al. *JAMA Network Open.*

eTable 2. Results of Multivariate Forward Linear Regression Quantifying the Contributions of Measured Factors to Difference in AAO of AD in Participants With Biomarker-Confirmed AD

Contributions of measured factors to ΔAAO	β	95% CI	p value
Intercept	11.47	-2.87, 25.81	0.12
Variables included in the model			
Female sex	-0.54	-4.58, 3.49	0.79
Education, mean years (±SD)	-0.53	-1.23, 0.18	0.14
Father affected	-9.04	-14.79, -3.30	0.002
Mother affected	-8.66	-15.46, -1.86	0.01
Parent with early-onset dementia	21.30	15.01, 27.59	<0.001
History of hypertension	3.11	-0.83, 7.05	0.12
APOE ε4/2 or *ε4/3*	5.01	1.02, 9.00	0.01
Symptomatic at study entry (retrospective determination of AAO)	-5.26	-9.64, -0.89	0.02

Information was available for all variables (no missing data)
AAO=age-at-symptomatic onset; ΔAAO=intergenerational difference in age-at-symptomatic onset; SD=standard deviation

© 2019 Day GS et al. *JAMA Network Open.*

eTable 3. Effect of Measured Variables on Participant AAO

Measured variable	Participant AAO, mean ± SD years		Mean Difference [±95% CI]	p value†
	Variable present	Variable absent		
Demographics				
Female sex (n=90)	70.2±8.5	71.6±8.0	-1.4 [-4.0, 1.1]	0.27
Education ≥16 years (n=89)	71.1±8.9	70.6±7.6	0.5 [-2.1, 3.1]	0.71
African American (n=19)	70.4±9.3	70.9±8.2	-0.6 [-4.6, 3.4]	0.78
Parental Factors				
Only mother affected (n=108)	71.7±8.0	70.1±9.8	1.5 [-1.7,4.8]	0.36
Only father affected (n=35)	70.2±9.8	71.7±8.0	-1.5 [-4.8, 1.7]	0.36
Both parents affected (n=21)	67.7±5.9	71.3±8.5	-3.6 [-7.4, 0.2]	0.06
**Parent with early-onset AD (n=9)**	**63.3±8.0**	**71.3±8.1**	**-8.0 [-13.5, -2.5]**	**0.004**
Acquired factors				
History of cardiovascular disease (n=51)	72.5±6.9	70.1±8.8	2.3 [-0.4, 5.1]	0.10
**History of depression (n=59)**	**67.6±7.1**	**72.7±8.4**	**-5.1 [-7.6, -2.5]**	**<0.001**
Body mass index >26.0 kg/m^2 (n=87)	70.0±8.1	71.9±8.5	-1.9 [-4.5, 0.6]	0.14
History of tobacco use >30 pack years (n=36)	71.8±7.1	70.6±8.6	1.2 [-1.9, 4.3]	0.45
History of alcohol abuse (n=10)	68.6±7.9	71.0±8.3	-2.4 [-7.7, 3.0]	0.38
**History of hypertension (n=81)**	**73.1±7.7**	**68.7±8.3**	**4.3 [1.9, 6.8]**	**0.001**
History of hypercholesterolemia (n=86)	70.7±7.4	71.0±9.2	-0.3 [-2.9, 2.2]	0.81
History of diabetes (n=16)	73.7±9.3	70.6±8.1	3.1 [-1.2, 7.4]	0.16
History of traumatic brain injury (n=15)	68.9±11.8	71.1±7.9	-2.1 [-6.6, 2.3]	0.34
**Symptomatic at study entry (retrospective determination of AAO)**	**69.1±8.9**	**73.8±8.9**	**-4.7 [-7.2, -2.1]**	**<0.001**
Heritable factors: APOE ε4 allele dose				
APOE ε4/2 or *ε4/3* (n=89) vs. no *ε4* (n=54)	71.0±7.8	73.1±8.5	-2.1 [-4.8, 0.7]	0.14
***APOE ε4/4* (n=21) vs. no *ε4* (n=54)**	**64.4±6.8**	**73.1±8.5**	**-8.6 [-12.4, -4.9]**	**<0.001**

Factors where p<0.05 are **bolded**.
†Student's t-test used for continuous variables; Chi square test for categorical variables, except those with expected values <5 (Fisher's Exact test)
AD=Alzheimer disease; AAO=age-at-symptomatic onset; SD=standard deviation

© 2019 Day GS et al. *JAMA Network Open.*

eTable 4. Multivariate Stepwise Linear Regression Quantifying the Contributions of Measured Factors to Participant AAO

Factors included in analyses	β	95% CI	p value
Intercept	78.34	71.06, 85.61	<0.001
Forced entry			
Female sex	-0.16	-2.56, 2.23	0.89
Education, mean years (±SD)	-0.25	-0.64, 0.14	0.21
Selected factors			
APOE ε4/4	-5.87	-9.25, -2.50	0.001
Active depression within 2 years	-4.54	-6.88, -2.19	<0.001
Father affected	-2.74	-5.07, -0.42	0.02
Symptomatic at study entry (retrospective determination of AAO)	-3.49	-5.85, -1.13	0.004
History of hypertension	3.93	1.64, 6.22	0.001
Excluded factors			
History of cardiovascular disease	0.13	--	0.06
Parent with early-onset dementia	-0.12		0.09
History of traumatic brain injury	-0.10	--	0.16
History of diabetes	0.05	--	0.44
Mother affected	-0.07		0.50
History of hypercholesterolemia	-0.03	--	0.69
History of alcohol abuse	-0.03	--	0.69
History of tobacco use (>30 pack years)	0.02	--	0.75
African American race	0.02	--	0.78
Body mass index (kg/m^2)	-0.02	--	0.82
APOE ε4/2 or *ε4/3*	-0.004	--	0.96

Model adjusted R^2=0.26; F=9.30; df=7; p<0.001
Information was available for all variables (no missing data)
AAO=age-at-symptomatic onset; SD=standard deviation

© 2019 Day GS et al. *JAMA Network Open.*

eReferences

1. Talbot C, Lendon C, Craddock N, Shears S, Morris JC, Goate A. Protection against Alzheimer's disease with apoE epsilon 2. *Lancet.* 1994;343(8910):1432-1433.
2. Cruchaga C, Kauwe JS, Mayo K, et al. SNPs associated with cerebrospinal fluid phospho-tau levels influence rate of decline in Alzheimer's disease. *PLoS Genet.* 2010;6(9):e1001101.
3. Cruchaga C, Del-Aguila JL, Saef B, et al. Polygenic risk score of sporadic late-onset Alzheimer's disease reveals a shared architecture with the familial and early-onset forms. *Alzheimers Dement.* 2018;14(2):205-214.
4. Lambert JC, Ibrahim-Verbaas CA, Harold D, et al. Meta-analysis of 74,046 individuals identifies 11 new susceptibility loci for Alzheimer's disease. *Nat Genet.* 2013;45(12):1452-1458.
5. Su Y, Blazey TM, Snyder AZ, et al. Partial volume correction in quantitative amyloid imaging. *Neuroimage.* 2015;107:55-64.
6. Schindler SE, Gray JD, Gordon BA, et al. Cerebrospinal fluid biomarkers measured by Elecsys assays compared to amyloid imaging. *Alzheimers Dement.* 2018;14(11):1460-1469.
7. Vlassenko AG, McCue L, Jasielec MS, et al. Imaging and cerebrospinal fluid biomarkers in early preclinical alzheimer disease. *Ann Neurol.* 2016;80(3):379-387.
8. Mishra S, Gordon BA, Su Y, et al. AV-1451 PET imaging of tau pathology in preclinical Alzheimer disease: Defining a summary measure. *Neuroimage.* 2017;161:171-178.

© 2019 Day GS et al. *JAMA Network Open.*
